# 磨玻璃样肺腺癌医患共同决策诊疗共识

**DOI:** 10.3779/j.issn.1009-3419.2026.106.06

**Published:** 2026-02-20

**Authors:** Xiaogang ZHAO, Jie CAI, Deping ZHAO, Chang CHEN, 磨玻璃样肺腺癌医患共同决策诊疗共识编写委员会

**Affiliations:** 200433 上海，同济大学附属上海市肺科医院; Shanghai Pulmonary Hospital, Tongji University, Shanghai 200433, China

**Keywords:** 肺肿瘤, 磨玻璃影, 磨玻璃样肺腺癌, 医患共同决策, 专家共识, Lung neoplasms, Ground-glass opacity, Ground-glass opacity-featured lung adenocarcinoma, Shared decision-making, Expert consensus

## Abstract

随着低剂量螺旋计算机断层扫描（low-dose computed tomography, LDCT）筛查的普及，磨玻璃影（ground-glass opacity, GGO）检出率持续上升。磨玻璃样肺腺癌多呈惰性生物学行为、预后良好，但其进展具有不确定性且各指南建议不一，使临床在“及时干预”与“避免过度诊疗”之间需要权衡，患者亦常出现随访焦虑与决策困惑。为在循证基础上规范全程管理并充分纳入患者价值观，本专家共识系统梳理GGO诊疗全过程，将医患共同决策确立为核心原则，强调结合患者价值观、生活目标与风险偏好共同制定方案。共识涵盖筛查起始与终止年龄、影像学诊断局限与侵入性诊断指征、手术指征与时机、亚肺叶切除与淋巴结处理策略、多发GGO管理、非手术替代疗法、术中冰冻病理可靠性、术后快速康复与疼痛管理、辅助治疗适应人群及随访策略等关键环节。通过推动多学科协作与结构化沟通，本共识旨在促进诊疗模式由“以疾病为中心”向“以患者价值观为中心”转变，提升医疗质量与患者满意度，减少过度诊疗，促进医患和谐。

## Abstract (en)

With the popularization of low-dose computed tomography (LDCT) screening, the detection rate of ground-glass opacity (GGO) has been continuously increasing. Ground-glass opacity-featured lung adenocarcinoma is mostly characterized by indolent biological behavior and favorable prognosis. However, its progression is uncertain and recommendations vary among guidelines. This places clinicians in a dilemma between timely intervention and avoiding overdiagnosis and overtreatment, while patients often suffer from follow-up anxiety and decision-making confusion. To standardize the whole-process management on an evidence-based basis and fully incorporate patient values, this expert consensus systematically reviews the entire procedure of GGO diagnosis and treatment, establishes shared decision-making between doctors and patients as the core principle, and emphasizes formulating management strategies by integrating patient values, life goals and risk preferences. The consensus covers key components including the starting and ending ages for screening, limitations of imaging diagnosis and indications for invasive diagnosis, indications and timing of surgery, strategies for sublobar resection and lymph node management, management of multiple GGOs, non-surgical alternative therapies, reliability of intraoperative frozen-section pathology, postoperative enhanced recovery and pain management, eligible populations for adjuvant therapy, and follow-up strategies. By promoting multidisciplinary collaboration and structured communication, this consensus aims to facilitate the transformation of the diagnosis and treatment model from disease-centered to patient value-centered, improve medical quality and patient satisfaction, reduce overtreatment, and promote doctor-patient harmony.

随着低剂量螺旋计算机断层扫描（low-dose computed tomography, LDCT）筛查普及，磨玻璃影（ground glass opacity, GGO）或磨玻璃结节（ground glass nodule, GGN）检出率显著升高^[1]^，已成为胸外科、呼吸科及肿瘤科等临床常见问题^[2]^。GGO为宽泛影像学征象，指肺内稍高密度影，边界可清晰或模糊，可见肺血管及支气管，无大小限制，GGN是GGO的特殊类型，特指薄层CT肺窗上类圆形、直径≤30 mm的GGO。所有GGN本质上均为GGO，仅满足“类圆形、直径≤30 mm”条件的GGO才属GGN范畴。此类病变具有与传统实性肺癌不同的生物学特性：生长缓慢、转移潜能低、预后较好^[3,4]^。其疾病谱系包括良性病变、非典型腺瘤样增生（atypical adenomatous hyperplasia, AAH）、原位腺癌（adenocarcinoma *in situ*, AIS）、微浸润腺癌（minimally invasive adenocarcinoma, MIA）及浸润性腺癌（invasive adenocarcinoma, IAC），其中病理确诊为腺癌的GGO即为磨玻璃样肺腺癌^[5]^。上述谱系特征为个体化、精准化管理提供重要依据，也使临床在干预时机与治疗强度选择上面临新挑战^[2]^。本共识主要探讨影像学表现为GGO的肺部病变管理，重点关注病理确诊为肺腺癌者（即磨玻璃样肺腺癌）。为便于表述并与多数临床研究及指南术语一致，下文涉及筛查、诊断、随访及部分治疗决策时，主要使用“GGO”这一涵盖性术语，特指经病理证实为腺癌的磨玻璃病变时，使用“磨玻璃样肺腺癌”。

当前GGO诊疗领域存在诸多尚未达成共识的争议焦点，贯穿疾病管理全程：（1）诊断层面：如何依据影像学特征[如大小、实性成分占比（consolidation-to-tumor ratio, CTR）、形态变化]精准预测病变浸润度与侵袭力?（2）治疗策略争议尤为突出：持续存在的纯GGO（pure GGO, pGGO），应手术干预还是积极随访?若手术，时机如何选择^[2]^?术式如何权衡（亚肺叶切除与肺叶切除）^[6,7]^?多发病灶应根治性切除还是选择性处理^[8]^?随访强度、频率及持续时间如何设定^[9]^?这些关键问题尚无全球统一“金标准”，不同学术流派及诊疗中心实践差异显著，构成临床决策“灰色地带”。这一决策困境并非GGO领域独有，在癌症防治中具有普遍性，如携带乳腺癌易感基因（breast cancer susceptibility gene, *BRCA*）突变个体面临预防性乳腺切除术抉择时，同样需在“早诊早治”与“过度治疗”间权衡科学证据与个体价值取向。

面对上述争议，传统“家长式”或单纯技术导向的医患沟通模式存在明显局限^[10]^。医生单方面依据指南或个人经验决策，或将复杂信息过度简化让患者被动选择，均难以应对GGO诊疗的高度不确定性、策略多样性及结局差异性。患者非被动接受者，其价值观、生活目标、疾病认知、风险耐受度、生活质量期望及对随访负担的态度，均为个体化决策重要因素。忽视患者价值取向的决策，即便技术层面“正确”，也可能导致满意度下降、依从性不佳甚至决策后悔^[11]^。

因此，医患共同决策（shared decision-making, SDM）在GGO诊疗这一高度依赖权衡的领域极具价值。SDM是结构化协作过程，医生需阐明疾病特征，系统呈现所有可行选项，结合证据分析各选项的潜在获益、风险及长期影响，患者充分理解信息后，表达其关切点、生活优先次序及价值偏好^[12]^，最终双方经沟通达成既符合最佳证据又契合患者个体情况的诊疗决策。将SDM融入GGO诊疗可优化决策质量，增强患者自主参与感与掌控感，改善医患沟通信任，提升治疗依从性及随访配合度。近期Chung等^[13]^发表于*Chest*的大样本回顾性研究显示，肺癌筛查中接受SDM的患者长期筛查依从性更高，筛查后第1年依从性提升26.5%，第4年差异扩大至32.5%，提示SDM有助于促进患者长期随访。SDM为“观察还是手术”“何时手术”“何种手术”等缺乏单一标准答案的问题，提供了可操作的决策框架。

《磨玻璃样肺腺癌医患共同决策诊疗共识》由同济大学附属上海市肺科医院多学科专家制定，旨在梳理诊疗关键环节与争议场景，明确SDM核心要素与实施流程，为临床提供可操作指导。本共识拟通过推广SDM，减少不同中心及学科间实践差异，助力医患在不确定性情景下开展结构化沟通与风险获益权衡，推动诊疗模式由“以疾病为中心”向“以患者价值观为中心”转变。本共识推荐的级别见表1。

**表1:** 共识推荐级别

推荐分级	含义
1A级	基于高水平证据（严谨的meta分析或RCT结果），专家组有统一认识。
1B级	基于高水平证据（严谨的meta分析或RCT结果），专家组有小争议。
2A级	基于低水平证据，专家组有统一认识。
2B级	基于低水平证据，专家组无统一认识，但争议不大。
3级	专家组存在较大争议。

RCT: randomized controlled trial.

## 1 筛查

### 1.1 筛查起始与终止年龄

GGO的筛查起始与终止年龄是临床重要议题，目前各指南推荐的筛查年龄尚不统一^[14]^。起始年龄争议集中于是否设统一界限及如何平衡筛查获益与风险，部分指南^[1,15]^建议45或50岁起始，亦有共识^[5,16]^提出40岁以上具备特定危险因素者筛查。这提示起始年龄非绝对标准，需结合患者吸烟史、家族史、环境暴露史及遗传背景综合判断。尤其非吸烟女性等特殊人群，应重视个体化风险评估，而非机械遵循年龄阈值。医患需充分沟通具体风险，共同商讨筛查必要性与时机，确保决策符合循证依据且尊重患者意愿。

筛查终止年龄的争议焦点，在于权衡老年群体筛查获益与潜在风险^[17,18]^。虽有指南推荐75岁为终止筛查上限，但更多观点支持按整体健康状况、预期寿命及治疗耐受性个体化判断^[9,14,17]^。高龄患者惰性GGO比例较高，筛查可能导致过度诊断与心理负担，故是否继续筛查需结合共存疾病、预期生存时间及个人意愿慎重评估^[18]^。尤其健康状况良好、预期寿命较长的老年人，不应仅因年龄剥夺筛查机会^[19]^。这一复杂决策需医患深入交流，明确筛查预期获益与可能风险，共同制定合理方案^[17,19]^。

青少年及年轻成人一般不纳入筛查，除非存在明确遗传高风险因素，此类情况需经多学科诊疗团队（multidisciplinary team, MDT）严格评估^[16]^。整个筛查决策过程中，SDM起核心作用^[13]^。医生需向患者阐明GGO惰性特性、筛查双面性及个体风险状况，帮助理解不同选择的依据与影响^[16]^，患者则充分表达担忧与价值取向，双方基于信任与合作形成共识性选择。该模式尤其适用于临界年龄或风险不明确的患者，助力在证据与偏好间找到最佳平衡。

**推荐意见1：建议吸烟、职业暴露、家族史等肺癌高危个体，从40岁起考虑低剂量CT筛查（2A），非吸烟女性等特殊人群，需结合二手烟、厨房油烟等暴露史个体化评估（2B）。75岁以上患者是否继续筛查，需结合健康状况、预期寿命及个人意愿综合判断，不宜仅以高龄终止（2A）。所有筛查决策均需医患充分沟通、共享信息后共同制定，确保筛查既符合患者最佳利益，也体现个人偏好与价值取向（2A）。**

### 1.2 筛查时间间隔

GGO筛查时间间隔是临床随访关键环节，尚无全球统一标准，争议核心在于平衡“及时发现病灶进展”与“避免过度检查及辐射、心理负担”^[20,21]^。首次筛查后复查时机存在分歧：有主张3-6个月短期复查排除一过性炎症，尤其针对混合型GGO（mixed GGO, mGGO）^[8,9]^，也有支持年度复查，认为pGGO多呈惰性生长，频繁检查获益有限^[20-23]^。长期稳定GGO是否坚持年度随访亦有争议，部分指南建议持续年度筛查，亦有证据支持稳定2年后适当延长间隔至1-2年，但均强调不可完全停止随访^[5,23,24]^。随访时长需结合患者年龄、整体健康及预期寿命综合判断，多数共识建议对GGO保持长期随访意识，因部分病变可能数年后才进展^[23,24]^。

临床制定筛查间隔需依托GGO类型、大小及动态变化等影像学特征，同时融入患者风险背景、心理状态和预期寿命等自身因素^[23,25]^。例如，实性成分多、直径大或有高危因素者通常需缩短复查间隔，多年稳定的微小pGGO可酌情延长^[23,25]^。此外，极度焦虑患者可酌情短期复查缓解忧虑，但需警惕过度检查，高龄或合并严重疾病者应采取更保守策略。技术一致性亦不容忽视，推荐固定机构与扫描参数保障随访可比性，复查建议采用标准剂量CT确保图像清晰，避免漏诊细微变化^[1]^。放射科医生需确保每次随访检查在层厚、剂量、重建算法等关键参数上一致，精准、可重复测量对比GGO，为后续决策提供可靠技术支撑。

SDM在本议题中起核心作用，医生应主动向患者阐释GGO惰性特点与随访双重性，既说明及时复查对早期发现进展的价值，也坦诚频繁检查可能的辐射暴露与心理压力^[3]^。制定随访计划时，须结合患者具体病情、风险层次、心理承受力及个人意愿个体化商讨，共同确定初始间隔与长期策略。Chung等^[13]^的研究进一步证实，肺癌筛查中接受SDM的患者长期筛查依从性更高，获益可持续多年，为SDM在GGO长期随访管理中的关键作用提供有力支持。每次复查后需重新评估并动态调整随访策略，尤其对随访间隔处于灰色地带的患者（如GGO长期稳定或年龄与预期寿命矛盾），更需充分沟通达成共识，体现“以患者为中心”的诊疗原则。

**推荐意见2：pGGO<5 mm者实施年度随访，稳定后可酌情延长至1-2年（2A），新发现或直径≥5 mm的pGGO建议6-12个月首次复查（2A），mGGO或实性成分≥5 mm者缩短至3-6个月复查（2A）。所有长期稳定GGO仍须坚持随访，间隔可适度延长但不建议终止（2A）。最终随访方案需基于GGO表现、患者风险、心理状态及预期寿命个体化制定，经医患充分沟通共同决策（2A）。**

GGO筛查SDM的临床路径图见图1。

**图1:**
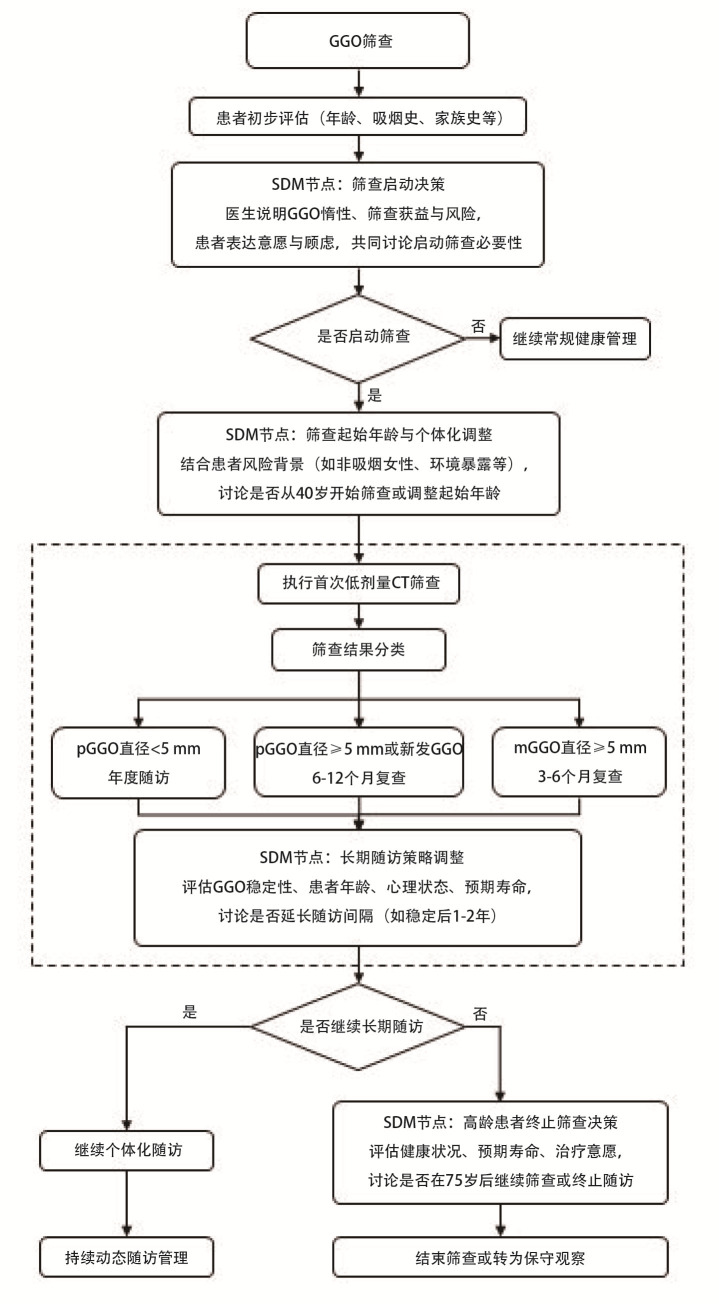
GGO筛查SDM临床路径图

## 2 诊断

### 2.1 影像学诊断的局限性

GGO影像学诊断虽是临床决策的重要依据，但其内在局限性显著影响诊疗策略的制定与医患沟通。高分辨率CT作为当前主要影像手段，对病变检出和随访不可或缺，却无法替代病理诊断，这一根本局限使其定性判断存在明显模糊性^[5]^。同一GGO影像既可能对应早期肺腺癌，也可能是炎症、出血或纤维化等良性病变，导致首次发现时多难以明确性质，引发立即干预或短期随访的争议，也带来患者焦虑与重复辐射暴露问题^[9,18]^。

即便影像高度疑似恶性，对病理亚型的预判仍有较大误差，尤其难以精准区分AAH、AIS与MIA，更可能低估病变实际浸润程度。近年研究^[26]^表明，CT值定量分析可一定程度辅助判断GGO病理阶段，如平均CT值>-472 HU常与IAC有关，较低值多见于前驱病变。但CT值受设备、重建算法及测量方法影响，判断阈值尚未统一，临床应用需谨慎解读。术后病理升级时有发生，直接影响手术范围确定和淋巴结清扫策略，成为术中决策与术后管理的争议焦点。此外，影像学对GGO生长速度、侵袭转移潜能等生物学行为的预测能力有限，存在评估盲区。正电子发射计算机断层显像（positron emission tomography, PET）/CT在实性成分少的GGO中诊断价值有限，淋巴结评估亦易出现假阴性，导致临床分期不确定，但在怀疑多发GGO转移或评估术后可疑复发等特定场景下，仍可提供重要代谢信息辅助综合判断。

GGO随访期影像评估同样面临测量误差与主观判断的挑战，大小、密度及实性成分变化的判定缺乏绝对客观标准，不同医师或不同时点测量可能存在差异，直接影响“进展”的定义与管理调整。对于多发GGO，影像在鉴别多原发与肺内转移、确定主导病灶等方面作用有限，需依赖分子病理等进一步技术手段，增加了临床决策复杂性^[27]^。面对这些局限，SDM尤为关键^[13]^。医生应主动向患者说明影像学结果的不确定性，包括诊断推断病理阶段的概率属性及随访中可能的误判风险，让患者理解诊疗选择的依据与权衡。放射科医生在MDT评估中需承担超越单纯描述的关键责任，应基于高分辨率CT影像特征（如大小、密度、形态、动态变化），结合CT值定量分析与定性评估，提供GGO性质（如良性可能性、浸润概率）及进展风险的初步判断，为临床决策提供直接影像学依据。

制定个体化方案时，需结合临床、影像及尽可能获取的病理信息综合判断，复杂病例强烈推荐MDT模式，集思广益弥补影像学不足^[28]^。同时，医生也需关注影像组学与人工智能等新兴技术在辅助诊断与预测中的应用前景，虽目前仍处探索阶段，但未来有望提升影像评估的客观性与预测精度。

**推荐意见3：推荐临床医师应用影像学评估GGO时，明确其在定性诊断、病理分级及生物学行为预测中的局限性，避免仅凭影像信息作出绝对判断（2A）。可结合CT值等定量指标辅助评估，但需注意其变异性与局限性（2B）。诊疗决策中，医生须与患者充分沟通影像结果的不确定性及对后续策略的影响，共同权衡不同方案的风险与获益（2A）。疑难病例建议通过MDT模式整合各类信息，提高诊断准确性与治疗合理性，所有决策应体现患者参与及个体化原则（2A）。**

### 2.2 侵入性诊断的必要性与风险

GGO是否需行侵入性诊断（如经皮肺穿刺、支气管镜活检），临床仍存显著争议，核心在于权衡明确病理诊断的获益与操作风险及对后续治疗的影响^[25]^。目前对适合手术的孤立性GGO，多数专家倾向于直接手术切除而非穿刺活检，因其兼具诊断与治疗作用，可一次完成、效率高，还能规避活检可能的假阴性、取样误差及气胸、出血、极少数针道种植等风险^[2,9,29-32]^。尤其GGO穿刺诊断面临独特挑战，包括病变密度低、质地软，常伴肺泡塌陷或纤维增生，穿刺针获取足够代表性组织的难度大，假阴性率高于实性结节（文献报道10%-30%），且病灶边界模糊、随呼吸移动度大，精准定位要求高，气胸等并发症风险不可忽视。此外，活检后的胸膜粘连或局部影像学改变，还可能增加后续手术难度或干扰病灶评估。

尽管如此，特定临床情境下侵入性诊断仍具重要价值^[33]^。影像学不典型、良性疾病（如炎症、感染）无法排除时，活检可为良恶性诊断提供参考信息，高龄、合并症多、功能状态差无法耐受手术，或患者坚决拒绝手术但需病理指导非手术治疗（如放疗、消融）时，活检亦属必要^[9,33]^。此外，多发GGO中具有不典型或侵袭性影像特征的病灶，活检可明确病理类型以指导整体治疗策略^[8]^，计划新辅助治疗时获取组织行分子检测是关键前提^[34]^。决定穿刺前需审慎权衡，小而纯的GGO或位置深、紧邻大血管/重要脏器的病灶，穿刺获益风险比较低，需更谨慎。

这一决策过程中SDM起核心作用^[13]^。医生应向患者清晰说明GGO的惰性特征，阐释直接手术与先行活检的利弊：前者效率高，但有一定手术创伤及潜在过度治疗风险，后者可能避免不必要手术，却伴随操作相关并发症、较高假阴性可能（尤其pGGO）及治疗延迟风险^[35]^。评估需综合考量：GGO可穿刺性（大小、位置、与血管距离）、拟用穿刺技术（如CT引导下同轴活检技术可提高取材成功率、减少胸膜通过次数）、操作者经验及病理科对少量活检组织的诊断能力。技术层面推荐用薄层CT精准规划路径，选择细针或组织芯活检针，操作中密切关注患者呼吸配合，最大化提高诊断率、降低风险。还需考虑患者整体健康状况及个人风险承受能力，充分尊重其对明确诊断或尽快治疗的偏好^[25]^；尤其临床判断处于灰色地带、患者对不确定性容忍度低时，更需充分沟通达成个体化选择^[10,13]^。

**推荐意见4：高度疑似恶性且适合手术的孤立性GGO，优先考虑直接手术切除，规避侵入性诊断的额外风险及诊疗延迟（2A），无法耐受手术、患者拒绝手术，或多发病灶需明确诊断指导治疗策略时，建议行侵入性诊断获取病理结果（2A）。所有决策需在全面评估病变特征、患者整体状况及意愿基础上，经医患充分沟通共同制定（2A）。**

GGO诊断SDM的临床路径图见图2。

**图2:**
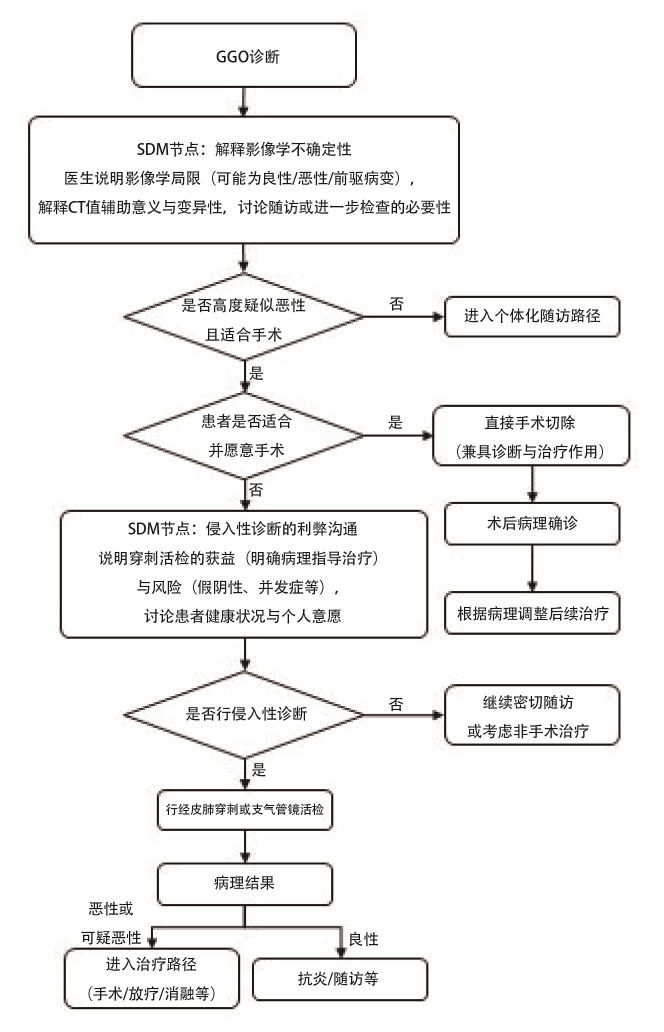
GGO诊断SDM临床路径图

## 3 治疗

### 3.1 手术指征与时机

GGO的手术指征与时机选择是核心争议，关键在于把握“转移成功之前的阶段”（病变获得局部或远处转移能力之前的生物学演进阶段，临床中用10年无复发生存（recurrence-free survival, RFS）率达到100%来评估此阶段），在积极干预与动态观察间取得合理平衡。部分观点支持疑似恶性GGO及时手术以规避进展风险及患者焦虑，Li等^[36]^对纯磨玻璃样肺腺癌术后10年生存研究证实，pGGO患者术后10年RFS率达100%，即使病理为IAC亦无复发，支持将pGGO视为可手术治愈的惰性亚型。长期随访研究^[3]^对135个pGGO中位随访193个月，发现即使GGO稳定10年后仍有3.9%会生长，提示惰性病变仍存进展可能。但更多证据^[37]^表明，多数pGGO及CTR≤0.25的mGGO具有显著惰性，处于“免疫平衡”状态，转移成功率极低。Li等^[38]^研究显示，直径≤2 cm且CTR≤0.25的肺部亚实性GGO，观察等待组5年无事件生存率达100%，与手术组（99.0%）呈非劣效结果（*P*<0.001），支持该人群采取保守策略。另一项针对1003个GGO（684例患者）的前瞻性研究^[39]^随访10年余，进一步证实随访组与手术组10年总生存率无显著差异，无论GGO稳定或增大，两组长期生存结局均无统计学差异，稳定与增大GGO的7年RFS率均达100%，且GGO增大不影响手术成本和时长。该研究明确CT随访可作为GGO适宜管理策略，直至出现实性成分。AIS、MIA及CTR=0的IAC切除后10年RFS率接近100%，代表典型“转移成功之前”阶段，即便随访中进展再手术，患者仍可获极高治愈率，提示存在充分“治愈窗口期”进行个体化决策^[36]^。

国内外指南对GGO处理差异显著，反映了不同国家对GGO生物学行为的认知不同^[25,40]^。肺结节诊治中国专家共识建议5-10 mm的pGGO随访增大至10 mm以上考虑手术^[9,41]^，日本指南将阈值提至15 mm，美国国立综合癌症网络（National Comprehensive Cancer Network, NCCN）指南推荐持续存在的pGGO直径≥20 mm才考虑切除，欧洲部分指南甚至放宽至30 mm^[40]^。这种差异源于人种特征、医疗资源、卫生经济学考量及对pGGO侵袭性评估的不同。需重视的是，影像学pGGO中MIA及IAC占比不容忽视，上海市肺科医院273例IAC回顾性研究^[42]^提示，对于密度较高的pGGO，即便直径<10 mm也应高度警惕浸润可能。Hu等^[43]^研究指出，结合pGGO大小、最大CT值及CT形态特征（分叶、毛刺、胸膜凹陷、空泡征、血管集束征等）构建的多参数预测模型，可显著提高IAC术前诊断准确性，为手术时机选择提供更精准依据。

持续存在的pGGO应在体积增大时手术还是等待实性成分出现?支持较早手术的观点认为，部分pGGO持续增大或实性成分增多可能提示侵袭性增强^[44]^。研究^[6,36]^显示，pGGO手术组10年RFS率为100%，随CTR升高复发风险显著增加。CTR>0.50且肿瘤>1 cm患者术后复发率达16.22%，且复发多集中于术后5年内，尤其周边型、可行亚肺叶切除的病灶，早期干预可能以较小创伤获得根本治愈。反之，主张谨慎随访的观点强调，实性成分出现是侵袭性增强的更可靠标志，过早手术可能增加过度治疗风险。需注意，气腔播散（spread through air spaces, STAS）风险随CTR升高而增加，CTR>0.50时发生率显著上升^[44]^，即便pGGO中也可观察到2%-11%的STAS，提示其存在一定生物学异质性，不能简单等同于“零进展风险”。因此，周边GGO且影像学高度怀疑浸润者可更积极干预；若为中央型且需肺叶切除，可严密随访至出现实性成分（CTR≤0.25），但应在淋巴结转移前完成干预。JCOG0201研究^[45]^对86例0.25<CTR≤0.50患者的分析显示其淋巴结转移风险增加，支持对CTR>0.25的mGGO采取更积极策略。欧洲心胸外科协会（European Association for Cardio-thoracic Surgery, EACTS）/欧洲胸外科医师学会（European Society of Thoracic Surgeons, ESTS）指南^[40]^强调应基于MDT评估，结合GGO动态变化、实性成分及患者风险因素综合决策。

影响手术决策的因素多样：GGO影像特征至关重要（直径、CTR及动态变化）^[25]^，通常直径较大（如>8 mm）、CTR>0.25或随访进展者更倾向手术^[25,44]^，上述IA期浸润性非小细胞肺癌（non-small cell lung cancer, NSCLC）研究^[44]^证实CTR>0.50是复发的重要预测因子，深分叶征、癌胚抗原（carcinoembryonic antigen, CEA）升高等也是预后不良的独立预测因子，也应充分评估^[46]^。患者年龄、预期寿命及心理状态同样关键，年轻患者可择期干预，高龄、合并症者宜随访，焦虑严重影响生活质量者，充分知情后可手术^[25]^。病理阶段预估直接影响策略，2018年《上海市肺科医院磨玻璃结节早期肺腺癌的诊疗共识（第一版）》提出，高度怀疑AIS且直径>8 mm的pGGO，若患者心理压力大可考虑手术，MIA和IAC则建议手术（MIA术后近100%治愈，IAC即使以GGO为主仍存小概率高危亚型或复发风险）^[4,26]^；多发pGGO常遵循“处理主病灶，随访次病灶”原则^[8,25,41]^。

这一复杂决策过程中SDM居核心地位。医生需清晰解释GGO的惰性特征、“转移成功之前阶段”及“治愈窗口期”等概念，结合证据说明病变通常进展缓慢，留有充分权衡时间^[3,36]^，客观阐述手术的潜在获益与风险、随访的意义与负担，在共同决策中充分讨论不同诊疗方案的风险获益^[25]^。最终方案应立足患者个体情况（GGO行为、年龄、健康状况、心理承受能力、个人价值观），通过充分沟通达成共识^[25,40]^。对于临界患者（如CTR约0.25），医患深入交流与共同选择尤为重要^[44]^。决策应保留适度弹性，尊重患者充分知情后的不同选择。当面临“手术”与“随访”取舍时，本质是在“早诊早治”与“避免过度治疗”间权衡，在根治可能性与长期管理策略间作出个体化选择^[36,40]^。

**推荐意见5：（1）持续存在的直径较大（通常>8 mm）、实性成分较高（CTR>0.25）或随访中进展（体积增大/实性成分增多）的mGGO，高度怀疑MIA或IAC者，建议活检或手术明确诊断（1B）。（2）10 mm及以上的pGGO，随访显示进展（体积增大/密度增加），或出现空泡（囊腔）、分叶、毛刺、支气管充气征、胸膜凹陷、血管聚集等恶性征象，高度怀疑MIA或IAC，且位于肺周边或单个肺段、可行亚肺叶切除者，倾向活检或手术明确诊断；若病变位于肺中央需行肺叶切除，可酌情继续随访至实性成分出现（CTR≤0.25）。持续存在（3-6个月）的15 mm以上pGGO，倾向建议活检或手术明确诊断（1B）。（3）10 mm以下的pGGO和8 mm以下的mGGO：①高度怀疑AIS者优先随访观察，若出现明显变化（直径增大>2 mm、密度显著升高、实性成分增加），则考虑缩短复查间隔或活检、手术明确诊断；②高度怀疑MIA或IAC者应行MDT讨论，医患共同协商个体化方案，若患者心理压力极大，可适当放宽活检或手术指征（2A）。（4）所有决策需结合患者年龄、预期寿命、健康状况、心理需求、病变预估病理阶段、病变位置等多方面因素个体化制定，在SDM框架下经充分沟通完成，尊重患者意愿并保留弹性空间。在保证肿瘤学安全的前提下，优先选择创伤小、肺功能保留多的治疗策略，避免过度治疗（2A）。**

### 3.2 手术方式选择

3.2.1 切除范围争议：亚肺叶切除与肺叶切除及淋巴结处理的个体化策略 GGO手术治疗策略是胸外科核心议题，关键在于将切除范围与淋巴结处理作为整体决策，而非独立环节^[40]^。现代GGO诊疗理念强调：基于肿瘤生物学特征，个体化同步确定切除范围与淋巴结处理策略，在肿瘤根治、肺功能保留及免疫功能维护间寻求最佳平衡^[9,37]^。

肺叶切除联合系统性淋巴结清扫作为传统标准术式，适用于实性成分多、浸润风险高或疑似淋巴结转移的病变^[25]^。但对于GGO为主型肺腺癌，其独特生物学行为促使我们重新审视该标准。研究证实，pGGO或CTR≤0.25的mGGO淋巴结转移率极低且预后良好。JCOG0804研究^[47]^显示，直径≤2 cm且CTR≤0.25病变亚肺叶切除后5年RFS率达99.7%。JCOG0802/WJOG4607L研究^[6]^表明，直径≤2 cm且CTR>0.50的周围型肺癌，解剖性肺段切除总生存不劣于肺叶切除。北美CALGB140503试验^[7]^进一步证实，直径≤2 cm周围型肺癌亚肺叶切除与肺叶切除总生存无显著差异。这些研究为亚肺叶切除应用提供坚实依据^[31]^。

但真实世界研究呈现更复杂的临床图景：美国胸外科医师协会数据库大规模研究纳入32,340例临床IA期（≤2 cm）NSCLC患者，发现肺叶切除、肺段切除的总生存及肺癌特异性生存均显著优于楔形切除，差异或与楔形切除淋巴结评估不足有关（中位取样仅3枚，Nx率达20.8%）^[48]^。首尔峨山医疗中心回顾1205例临床IA期肺腺癌患者发现，保证充足切缘（cT1a≥1 cm或≥肿瘤直径，cT1b≥2 cm或≥肿瘤直径）且充分淋巴结评估（≥3个N2站+≥1个N1站）前提下，楔形切除与肺段切除患者的RFS率无显著差异（5年RFS率：92.4% *vs* 93.3%）^[49]^。这些发现强调，无论何种切除方式，充足切缘与系统性淋巴结评估至关重要。

淋巴结处理策略需与切除范围同步个体化制定。Zhang等^[37]^III期随机对照研究（ECTOP-1009, NCT04527419）中期分析显示，CTR≤0.50的GGO为主型IAC患者中，系统性纵隔淋巴结清扫未发现转移，且清扫组手术时间、出血量、术后住院时间均显著增加，还出现乳糜胸、术中大出血等严重并发症，提示此类患者常规系统性清扫难获生存获益，反而增加创伤与并发症风险。纳入400例手术切除pGGO的多中心回顾性研究（GORDON研究）^[50]^通过受试者工作特征（receiver operating characteristic, ROC）曲线分析，明确pGGO侵袭性放射学预测指标：直径≥18 mm且CT值>-390 HU联合预测准确率达81.2%，曲线下面积（area under the curve, AUC）为0.874，可为术前风险分层及手术方案优化提供参考；而该研究核心结果显示，所有纳入的pGGO患者（含346例IAC）均未发现淋巴结转移，5年淋巴结RFS率达100%，提示pGGO无论是否具备高侵袭性放射学特征，均无需常规行系统性淋巴结清扫。此外，淋巴结是T细胞活化与免疫调控关键场所，2023年*Cell*报道的研究^[51]^提示免疫治疗相关应答T细胞主要源于淋巴结，过度清扫可能削弱免疫应答，因此无转移证据时，应在肿瘤学安全前提下尽量避免不必要清扫，兼顾转移控制与免疫功能保留。

基于上述证据，临床决策遵循以下原则：直径≤2 cm、CTR≤0.25的GGO，行亚肺叶切除（楔形或肺段）且不做系统性淋巴结清扫或仅采样是合理的^[37]^，中央型需在三维重建辅助下保证足够阴性切缘（通常≥2 cm或≥肿瘤直径）^[40]^。直径≤2 cm且CTR>0.50的病变，解剖性肺段切除推荐为标准选项之一，但需注意10%-20%可能存在高危病理亚型（如微乳头/实体型成分>5%）或STAS，需考虑扩大切除及淋巴结清扫范围^[6]^。术中冰冻病理诊断准确性是联合决策关键：提示AAH、AIS或MIA，可行亚肺叶切除且不清扫或仅采样淋巴结；贴壁样为主型IAC且影像学CTR≤0.50，可行亚肺叶切除联合叶特异性淋巴结清扫或采样；含高危亚型IAC或CTR>0.50，应行肺叶切除联合系统性淋巴结清扫。多发GGO需根据主病灶病理结果决定切除范围与淋巴结策略。

这一综合决策中SDM至关重要。医生需向患者详细说明不同术式的核心利弊：肺叶切除根治性强但创伤大、对肺功能影响显著；亚肺叶切除可保留更多肺组织，利于后续生活质量及应对多发病变，但可能伴随略高的局部复发风险^[6,7]^。同时结合患者意愿、生活质量期望及复发风险承受能力个体化讨论。所有决策须结合术前高分辨率CT评估、术中冰冻病理结果及患者肺功能、年龄、合并症，经医患充分沟通制定。建议在具备三维重建、胸腔镜/机器人手术平台及术中病理快速诊断的医疗中心开展个体化手术，加强MDT协作以降低决策不确定性。

**推荐意见6：直径≤2 cm的GGO，CTR≤0.25可行楔形或肺段切除，0.25<CTR≤0.50行肺段切除，淋巴结行选择性清扫或仅采样（1B）。直径≤2 cm且CTR>0.50的病变，推荐解剖性肺段切除或肺叶切除，依据术中冰冻病理决定淋巴结处理范围：高危病理亚型或CTR>0.75，建议肺叶切除+系统性淋巴结清扫；贴壁样为主型且CTR为0.50-0.75，可行肺段切除+叶特异性淋巴结清扫。直径≤2 cm、CTR>0.50且肿瘤位于胸膜正下方（边缘距离<1 cm）的病变，技术上可保证足够切缘时，肺功能储备不足或基础疾病多的患者，楔形切除可作为备选项，但需至少3站纵隔淋巴结采样（1B）。病灶较大（>2 cm）、实性成分多（CTR>0.50）、位置深或疑似淋巴结转移者，优先考虑肺叶切除+系统性淋巴结清扫（1A）。所有手术方案须依据术前CT评估、术中冰冻病理及患者个体情况，经医患充分沟通共同决策（2A）。**

3.2.2 微创术式优化：技术选择与成本效益 GGO微创手术目前以胸腔镜技术为核心，多种术式并存，主要包括单孔胸腔镜手术（uniportal video-assisted thoracoscopic surgery, U-VATS）、机器人辅助胸腔镜手术（robot-assisted thoracic surgery, RATS）及多孔胸腔镜手术（multiportal video-assisted thoracoscopic surgery, M-VATS），各有技术特点与适用场景^[52,53]^。U-VATS技术已经成熟，单一切口创伤小、美观性好，适用于绝大多数GGO手术，尤其适合周围型病变楔形切除或简单肺段切除；复杂肺段切除等精细操作，若术者经验丰富，亦可考虑应用U-VATS，但操作难度高、存在器械干扰，对术者经验要求较高^[54,55]^。RATS依托三维高清视野、器械灵活运动及震颤过滤等优势，在复杂肺段切除、淋巴结清扫等精细操作中表现突出，缺点是设备与手术成本高，且缺乏触觉反馈^[52,56]^。M-VATS应用最广泛、技术最成熟，操作稳定且适应性强，可覆盖绝大多数胸腔镜手术，但多切口设计在术后疼痛与美观上略逊于单孔^[55,57]^。

确定具体手术方式需综合多方面因素：病变特点与手术复杂度是关键，简单切除可根据术者熟练度及患者意愿选择U-VATS或M-VATS，复杂解剖性切除更适合RATS或M-VATS^[58]^。外科医生对不同术式的掌握程度也起决定作用，U-VATS学习曲线陡峭，RATS需系统培训，M-VATS技术普及度最高^[53]^。此外，还需考量患者诉求，如术后疼痛控制、恢复速度、美观要求及经济承受能力，年轻或费用敏感患者可能倾向U-VATS或M-VATS，部分患者为追求精准性可接受RATS^[56]^。医院设备条件同样制约选择，RATS依赖高端机器人系统，并非所有单位均可开展。

这一决策过程中SDM居核心地位。医生应客观全面向患者介绍各术式优缺点，包括创伤大小、疼痛程度、恢复周期、费用差异及特定病情下的适用性，并结合自身经验给出专业建议，同时需充分尊重患者意愿与价值取向，如对微创程度的期望、经济成本考量及对手术安全性与效果的权衡。最终目标是结合患者病变特征、身体条件、心理需求及医疗资源，共同确定最合适的手术方案，在保障肿瘤根治的前提下，最大限度减少创伤、促进康复并提升治疗体验^[10,54]^。

**推荐意见7：根据GGO病变位置、大小、复杂程度及患者个体情况综合选择微创手术方式：U-VATS技术已趋成熟，适用于绝大多数GGO手术；周围型小型病变或简单术式优先考虑U-VATS；复杂解剖性切除推荐RATS或M-VATS，术者经验丰富时亦可选用U-VATS；最终决策需结合医院设备条件、外科医师技术特长及患者对创伤、康复、费用等的意愿，经医患充分沟通共同制定（2A）。所有手术均应在保证肿瘤学根治的前提下，追求最大程度微创与快速康复。**

### 3.3 多发GGO的处理策略

多发GGO处理是胸外科临床难点与热点，核心在于准确鉴别多原发肺癌与肺内转移，在此基础上制定以根治为目标、最大化保留肺功能的个体化治疗方案^[8,25,27]^。Kim等^[59]^多中心队列研究显示，409例手术切除的亚实性肺腺癌患者中，共检出1791个同步亚实性GGO，平均随访80.6个月，仅16.9%出现生长，7.9%最终诊断为肺癌，且均为0-I期腺癌，未显著影响患者总体生存。该研究进一步指出，GGO进展主要与自身影像学特征有关（如部分实性类型、直径较大、气泡透明征或胸膜牵拉等），数量并非关键因素。这与《多发磨玻璃结节样肺癌多学科诊疗中国专家共识（2024年版）》^[8]^观点一致，认为多发GGO样肺癌是多原发肺癌特殊亚型，各病灶为“独立个体”而非转移灶，预后良好，总体预后取决于主病灶^[41]^。

临床主要依据高分辨率CT影像特征（形态、密度、分布、动态变化等）初步判别，如GGO位于不同肺叶、形态各异、生长速度不一致，多倾向多原发，但最终需结合分子病理确诊。尽管基因测序可提供重要依据，但因成本及肿瘤异质性等问题，临床推广受限。术后或活检病灶间无相同驱动基因突变，是多发GGO样肺癌明确诊断标准之一。

需注意的是，我国多发GGO检出率已达20%-40.5%，且呈年轻化趋势，70岁以上人群检出率为35%-37.52%。除影像评估外，可结合实验室检查，包括线粒体相关酶[超氧化物歧化酶（superoxide dismutase, SOD）、天门冬氨酸氨基转移酶（aspartate aminotransferase, AST）]、细胞因子[白细胞介素-6（interleukin-6, IL-6）、肿瘤坏死因子-α（tumor necrosis factor-α, TNF-α）]、免疫指标[CD4/CD8、自然杀伤（natural killer, NK）细胞]、肿瘤标志物[CEA、细胞角蛋白19片段（cytokeratin 19 fragment, CYFRA21-1）]等综合判断，提高鉴别准确性。

治疗策略已从“根治性切除”转向“保全肺功能前提下有效控制疾病”^[25]^。Kim等^[59]^研究亦支持此策略，指出同步GGO生长不显著影响生存，因此主病灶通常建议积极处理，根据大小、CTR及位置选择肺段或楔形切除以保留健康肺组织，仅主病灶符合肺叶切除指征时考虑肺叶切除。pGGO手术指征可适当从严，如干预阈值放宽至15 mm^[41]^。次病灶以定期随访为主，明确进展后再考虑干预。同期或分期手术需综合考量病灶分布、患者肺功能及耐受程度，双侧病灶尤其肺功能储备较差者，更适宜分次手术，避免术后肺功能严重受损，肺总切除范围不超过10个肺段^[41]^。这与《多发磨玻璃结节样肺癌多学科诊疗中国专家共识（2024年版）》^[8]^手术原则一致：优先处理主病灶、兼顾次病灶，在符合肿瘤学原则及保留肺功能前提下尽可能同期切除所有病灶，但多数情况下无需切除全部GGO，主病灶以肺叶或肺段切除为主，次病灶行肺段或楔形切除，同侧次病灶位于肺外2/3可同期亚肺叶切除，位于肺内1/3或对侧、实性成分<5 mm或CTR<0.25的病灶可密切随访或热消融^[41]^。淋巴结处理方面，GGO为主型病灶通常仅对主病灶采样或叶特异性清扫，不推荐常规系统性清扫^[37]^。术前推荐MDT讨论，整合多学科专家意见，结合人工智能辅助评估及患者意愿制定方案^[8]^。

非手术治疗在多发GGO管理中地位上升，尤其消融技术具一定优势。立体定向体部放疗（stereotactic body radiation therapy, SBRT）、消融等技术为无法耐受手术、次病灶进展或拒绝再次手术者提供可行选择。《多发磨玻璃结节样肺癌多学科诊疗中国专家共识（2024年版）》^[8]^提出，影像引导下热消融（image-guided thermal ablation, IGTA）是多发GGO样肺癌首选治疗方式之一，或者是手术切除的重要补充，手术联合IGTA（杂交手术）是新术式^[41,60]^。但其用于可手术患者次病灶处理仍存争议，需严格掌握适应证，结合病灶大小、位置、病理类型等综合判断^[41]^。此外，循环肿瘤DNA（circulating tumor DNA, ctDNA）、外泌体miRNA等无创评估技术，未来有望在风险分层与疗效监测中发挥重要作用。

整个诊疗过程中SDM居核心地位。医生需帮助患者理解，如Kim等^[59]^研究指出，多发GGO通常进展缓慢，可视为需长期管理的“慢性病”，治疗目标为控制进展、消除主要威胁、维持生活质量，而非追求影像学完全清除。应结合患者年龄、肺功能、心理状态及个人意愿制定方案，明确动态随访的重要性^[21]^。即使术后，仍需对残留或新发病灶终身监测，随访策略可参考肺部影像报告和数据系统（Lung Imaging Reporting and Data System, Lung-RADS）分类及临床肿瘤概率模型实施分层管理^[1,3]^。

**推荐意见8：多发GGO应在MDT讨论基础上，结合病灶影像学特征、动态变化、病理类型及患者个体情况制定个体化策略（2A），MDT团队需包含胸外科、呼吸科、肿瘤科、放疗科、影像科及病理科医生。主病灶建议行亚肺叶切除（根据大小、CTR及位置选肺段或楔形切除），必要时行肺叶切除（如病灶过大，CTR过高，亚肺叶切缘不足，冰冻病理提示高危亚型、STAS等不利因素等），次病灶以定期随访为主（2A），同期手术需严格评估肺功能及手术创伤，避免同期双侧肺叶切除（2A）。淋巴结处理以主病灶影像特征及术中冰冻病理为依据，无需常规系统性清扫（2B）。不宜手术的病灶可考虑SBRT或消融等非手术治疗，其中消融技术处理多发GGO具一定优势（2B）。所有决策需经医患充分沟通共同制定，突出长期管理、肺功能保全及生活质量维护的核心目标（2A）。**

### 3.4 非手术替代疗法的地位

GGO诊疗中SBRT、消融等非手术替代疗法有明确特定地位^[15]^。SBRT通过多角度高精度聚焦照射，单次给予消融性高剂量（通常4-8次完成疗程）^[61]^，借助剂量快速跌落技术保护正常组织，具有无创、无出血或气胸风险特点，局部控制率较高（不可手术患者5年局部控制率达93%）^[62]^，但需注意远期诱发二次肿瘤风险，尤其青少年患者需慎重^[63]^。消融属微创操作，主要包括射频消融（radiofrequency ablation, RFA）、微波消融（microwave ablation, MWA）、冷冻消融（cryoablation, CA），经皮穿刺产生热或冷效应直接毁损肿瘤，能较好保留肺功能且可重复实施，尤其处理多发GGO具有一定优势，但存在气胸（发生率10%-60%）、出血等穿刺相关风险^[60,64]^。两者均无法有效获取病理标本用于诊断及分子分析，此为固有局限^[65]^。

SBRT已成为不可耐受手术（如心肺功能差、高龄、合并症多）或拒绝手术的早期周围型NSCLC患者的标准替代根治方案^[15]^。应用需严格把握适应证（通常为T1-2N0M0，直径≤5 cm），中央型病灶（近端支气管树2 cm内）需特别谨慎，采用更保守分割方案（如5-9 Gy/次）以降低支气管瘘、大出血等严重风险。消融技术各有特点：MWA能量高、升温快，更能克服“热沉效应”，适于较大病灶或近血管者；CA镇痛效果好且或可激发免疫应答，适于胸膜旁病灶；RFA应用最经典。消融成功关键在于保证消融边界（消融后需超出GGO肿瘤边缘至少5-10 mm）^[60,66]^。

但对于多发GGO，两种技术在处理对侧或同侧次病灶、避免多次手术创伤方面具独特价值。但身体条件适宜手术的患者，目前缺乏高级别证据[如大规模III期随机对照试验（randomized controlled trial, RCT）]证明非手术疗法与手术长期生存等效，因此手术仍是首选金标准^[15]^。尽管热消融在部分场景（如肺叶内、明确进展但患者无法/不愿立即肺叶切除的病灶）可作为延缓手术、保留肺功能的过渡或替代选择，但其临床应用仍存争议，国内外指南均严格限制其作为首选方案^[60]^。ROSEL/STARS等研究^[67]^虽显示SBRT有积极结果，但证据级别有限，两者在此群体中应用应视为探索性选择。选择SBRT或消融需经MDT综合评估：中央型或较大病灶更倾向SBRT，周围型或小病灶（尤其≤3 cm）可考虑消融，尤适用于希望一次完成治疗、费用敏感或需处理多个分散病灶的患者，需充分考虑本地技术条件与经验^[68]^。

肺叶内中央型进展病灶的临床决策尤为困难^[59]^。传统策略包括继续随访至符合肺叶切除指征再手术，或对进展灶先行消融，复发后再考虑手术。后者虽可能为部分患者争取肺功能保留机会，但缺乏远期疗效数据支持，须谨慎采用^[60]^。此时更应强调SDM，结合病灶特征、进展速度、患者肺功能基线及个人意愿，全面权衡消融、手术及主动监测的利弊。

整个决策过程中SDM居核心地位。医生需全面客观地向患者介绍手术、SBRT、消融及主动监测等策略的利弊，包括手术的根治优势与创伤风险、非手术疗法的微创特点与不确定性（如无法有效获取病理、长期数据有限、SBRT对可手术者的争议性、消融的穿刺风险及疗效评估特殊性等），帮助患者依据自身病情、价值观及生活质量要求做合理选择^[62]^。尤其临界状态或存在多种可能性的患者，更需充分沟通明确治疗目标与预期，共同制定个体化方案。治疗后须长期规律随访，采用增强CT（消融后需注意“煎蛋征”“蛋壳征”等特有影像演变规律）或PET/CT（建议SBRT后6个月进行）等手段，及时评估疗效并监测复发或新发病变^[60]^。

**推荐意见9：SBRT和消融作为GGO非手术替代疗法，主要适用于不可耐受手术或拒绝手术的早期肺癌患者，及多发GGO次要病灶（主要病灶首选手术）的处理（2A）。SBRT是不可手术早期肺癌的标准选择，具体剂量和分割方案需根据结节大小和位置进行选择（2A）。消融技术选择（RFA、MWA、CA）需根据病灶特点（大小、位置、与血管关系）、设备可用性及操作者经验个体化决定，确保足够消融边界（2B）。肺叶内中央型进展、需保留肺功能或暂不宜手术者，经MDT评估及医患充分共同决策后，可审慎消融作为过渡处理（2A）。若非手术方案纳入考量，须经MDT评估并充分告知患者各自风险获益及不确定性后，审慎选择SBRT或消融作为过渡处理（2A）。所有决策需医患共同参与制定，确保患者充分知情并兼顾疗效与生活质量，治疗后须长期规律随访（2A）。**

### 3.5 术中冰冻病理（frozen section, FS）的可靠性

FS是GGO手术决策的关键，也是外科与病理科的重要会诊环节^[69]^。其价值在于30 min内快速评估切除标本，为术者明确病变性质、指导切除范围及淋巴结处理方案提供即时依据^[70]^。但诊断可靠性存在固有局限，主要体现为术中冰冻与最终石蜡病理的不符率，国内外报道总体一致率超95%，而GGO的“诊断升级”（冰冻报MIA或AIS，石蜡病理确诊IAC）尤为值得关注，发生率为5%-15%^[71]^。

这种差异源于三方面：一是取材误差，病理医生需数分钟内肉眼选取标本“最重”病变区域，小而形态不规则的GGO精准定位浸润灶难度大；二是制片技术局限，冰冻切片易受冰晶伪影、组织皱褶（肺组织柔软更易发生）等影响，切片质量（甲、乙、丙三级）直接关联诊断清晰度；三是诊断判读难度，时间压力下区分AAH、AIS、MIA及贴壁型IAC等形态学差异细微病变，存在主观性和“灰区”^[69]^。需注意，研究^[72]^显示<2 cm、CTR<0.50的GGO，即使约6.3%出现术中低估（升级为IAC），患者淋巴结转移率仍极低，5年RFS率达100%，提示此类诊断升级通常不影响良好预后^[72]^。

这种不确定性引发临床决策争议：一方主张完全信任FS结果，对影像学符合GGO优势型、直径≤2 cm且FS报AIS/MIA的病灶，仅行亚肺叶切除且不清扫淋巴结，依据是术后即使升级，亚肺叶切除远期疗效足够，可通过密切随访管理不确定性；另一方更谨慎，建议即使FS结果良好，仍考虑解剖性肺段切除（保证切缘）或淋巴结采样^[6,7]^。当前共识更倾向前者，尤其在JCOG0804等高级别证据支持下，特定条件GGO病变采取限制性切除及有限淋巴结处理已获广泛认可^[31]^。

术后石蜡病理升级是否需二次手术补做淋巴结清扫仍存争议^[40]^。主流观点^[37]^认为，初次未行淋巴结清扫、最终病理为贴壁样为主型IAC且影像学无淋巴结转移证据者，首选密切随访而非预防性二次手术，因二次手术创伤可能大于潜在获益。

提升FS可靠性可采取多项策略：术中对可疑区域充分取材、加强外科与病理医生实时沟通（尤其结合术前CT影像特征如CTR综合判断）、联合术中细胞学印片等技术互补。诊疗全程中SDM居核心地位。医生术前需向患者详细说明FS的作用与局限性，明确告知诊断升级可能性及通常良好的预后意义，共同商讨不同情景的应对策略。患者需理解随访重要性，参与制定个体化手术方案，在微创与肿瘤学安全性间达成平衡。

**推荐意见10：FS病理应作为GGO手术决策的重要参考，但非唯一依据（2A）。FS提示AIS或MIA，行亚肺叶切除及免淋巴结清扫为合理标准方案（1B）；术后石蜡病理升级为贴壁为主型IAC，无淋巴结转移临床或影像学证据者，首选密切随访而非二次手术（2B）。所有决策需结合术前高分辨率CT特征、术中所见及病理结果综合判断，术前与患者充分沟通冰冻诊断的潜在不确定性及各类预后情景，共同制定手术及术后随访计划（2A）。**

GGO治疗SDM临床路径图见图3。

**图3:**
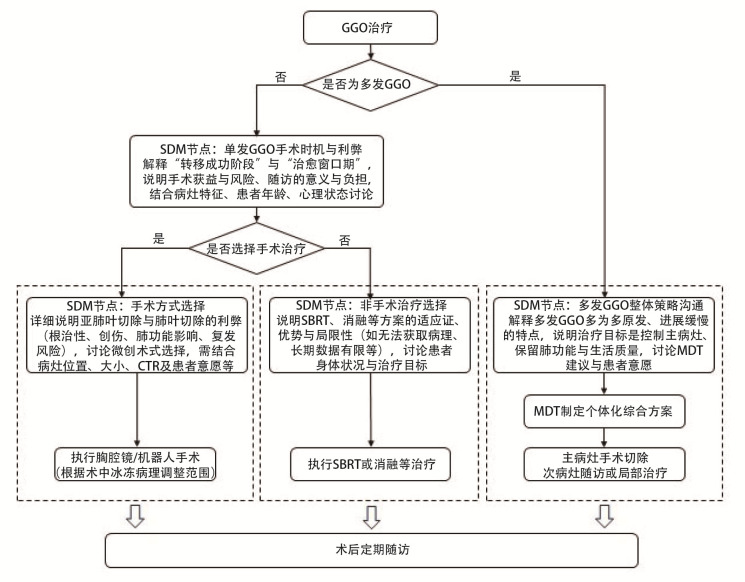
GGO治疗SDM临床路径图

## 4 术后康复

### 4.1 快速康复流程（enhanced recovery after surgery, ERAS）的个体化制定

ERAS理念在GGO围术期的个体化应用，是提升手术安全性与康复质量的重要途径^[73]^。核心是结合GGO病理惰性强、淋巴结转移率低、多采用亚肺叶切除保肺术式的特点，针对性调整ERAS流程，在保障根治效果的同时，最大程度减少创伤、缓解应激、加速恢复^[74]^。鉴于GGO患者早期肺癌占比高、高龄合并症者增多且微创手术（VATS/RATS）为主流，全面实施ERAS可实现住院时间缩短30%-50%、并发症减少的目标^[53]^。

个体化ERAS方案需综合多因素制定^[74]^。病变特征层面，小型周边型GGO、FS提示AIS/MIA者，可推行激进微创策略，优先U-VATS，力争术中“无管化”（不留胸管或用细管）或早期拔管<450 mL/24 h即可），避免常规放导尿管，最大化器官微创与快速康复；需肺叶切除或淋巴结清扫的浸润性癌，则在彻底切除基础上优化镇痛与恢复措施。患者因素同样关键，需全面进行术前心肺功能评估[如预计术后第一秒用力呼气容积（predicted postoperative forced expiratory volume in one second, ppoFEV_1_）/预计术后一氧化碳弥散量（predicted postoperative diffusing capacity of the lung for carbon monoxide, ppoDLCO）、心肺运动试验测最大摄氧量（maximal oxygen uptake, VO_2_max）]，年轻体能好者可适用日间手术及激进康复计划，高龄、合并症多或高危（吸烟史≥400年支、临界肺功能）者，需强调术前预康复（营养支持、戒烟≥4周、体育康复训练），谨慎调整ERAS措施，优先保障安全与耐受^[75]^。近期前瞻性随机对照研究^[76]^支持按患者风险分层实施多梯度个体化ERAS，依手术复杂度、患者基线状况与合并症划分康复强度等级，在安全前提下最大化康复效益。

麻醉与术中管理直接影响ERAS效果。推荐肺保护性通气，联合区域麻醉（如椎旁阻滞）与全身麻醉的多模式镇痛[常规用对乙酰氨基酚和非甾体抗炎药（nonsteroidal anti-inflammatory drugs, NSAIDs）]，减少阿片类依赖^[77]^；实施目标导向液体管理（首选平衡晶体液，避免过量输注）^[78]^；采取主动保温及多模式措施预防术后恶心呕吐^[79]^。这些措施可减少手术应激，为早期下床、进食奠定基础。

术后管理聚焦加速康复核心环节^[80]^：（1）疼痛管理需精准，依托区域镇痛减少阿片类对胃肠功能的影响，联合物理疗法促恢复；（2）鼓励术后24 h内下床，尽早恢复经口进食（术前2 h可进清液，术后当天进食）；（3）强化肺康复训练（如激励式肺量计吸气训练、功率自行车或爬楼梯训练），改善呼吸功能与运动耐力^[80,81]^。GGO患者预后卓越（如AIS/MIA 5年RFS率近100%），术后随访可适当简化（如术后2年内每年1次），无需常规行头颅磁共振成像（magnetic resonance imaging, MRI）、骨扫描或PET/CT，可借助远程医疗管理，契合“手术在院、康复在家”的快速康复路径^[82]^。

围术期全程SDM是个体化ERAS的关键。医生需充分沟通患者病变特点、拟行术式及适配的康复路径，帮助理解GGO惰性、“转移成功之前阶段”与“治愈窗口期”概念，合理设定康复期望；尊重患者对疼痛控制、恢复速度及生活质量的诉求，共商术前预康复、术中麻醉与术式选择、术后镇痛及早期活动等具体措施。ERAS成功实施需MDT协作，胸外科、麻醉科、营养科、康复科及护理团队共同制定执行全流程优化方案，通过持续沟通与动态评估，优化康复路径，提升治疗依从性与整体满意度。

**推荐意见11：推荐将ERAS理念全面融入GGO围术期管理，依据病变特征（分期、CTR）、手术方式（亚肺叶/肺叶切除）及患者情况（年龄、肺功能、合并症）实施个体化优化。早期GGO强烈推荐微创术式、精准淋巴结处理、激进管道管理（早期拔管/无管化）及强化术后肺康复；复杂手术或高危患者需在保障安全前提下调整ERAS措施，强调术前预康复。所有ERAS方案须经SDM制定，在MDT协作下执行，确保患者充分理解并参与康复，实现减少创伤、降低并发症、缩短住院时间及改善治疗体验的目标（2A）。**

### 4.2 呼吸康复的必要性与强度分层

呼吸康复在GGO患者围术期管理中具有重要地位，是ERAS理念的关键组成^[83]^。核心价值在于通过系统功能训练与健康指导，有效降低术后肺部并发症风险，改善肺功能、运动耐量及生活质量，缓解疾病与手术相关心理压力^[84]^。因患者年龄、基础肺功能、运动能力、营养状态及手术范围差异较大，呼吸康复方案需强调个体化，依据术前评估科学分层设定强度，实现安全与有效统一。

根据术前评估指标（年龄、肺功能损害程度、运动耐量、营养状况、吸烟史、呼吸肌力量等），可划分不同康复强度层次：高龄、肺功能中重度受损、运动耐力低或营养不良者，术前建议2-4周中高强度康复训练以提升手术耐受性；肺功能基本正常或轻度受损、体能较好者，1周左右预康复即可^[75,85,86]^。运动形式应涵盖有氧训练、抗阻训练、呼吸肌专项训练，结合气道廓清技术、早期下床活动及健康教育与心理支持，形成多维度干预体系^[83,85,86]^。全程需密切监测患者反应，避免过度疲劳，识别发热、血小板严重降低或肿瘤骨转移等禁忌证，保障康复安全。

SDM对呼吸康复的制定与实施至关重要^[87,88]^。医生需向患者详细说明呼吸康复的意义、预期获益及潜在风险，帮助其认识到对术后恢复、减少并发症的积极作用^[85]^，同时充分了解患者体力状况、个人意愿及实施条件，共商可行的康复目标与计划。例如，年轻患者若期望尽快恢复日常活动，可适当提升运动强度；高龄、合并多种疾病且耐受性差者，应以安全为首要原则，采用循序渐进方案^[89]^。通过持续沟通与动态调整，建立医患信任合作关系，提升康复依从性与整体效果。

**推荐意见12：建议所有手术GGO患者接受个体化、分层次呼吸康复干预。术前依据年龄、肺功能、运动耐量、营养状态等评估康复需求，给予1-4周中高强度预康复训练，术后尽早下床，结合有氧、抗阻训练及呼吸技巧训练促进功能恢复。康复方案须经医患共同讨论制定，兼顾科学性、安全性与可执行性，实施中保持沟通调整，最大程度改善围术期体验与远期生活质量（2A）。**

### 4.3 术后疼痛管理策略

GGO术后疼痛管理是ERAS路径关键环节，策略已从药物依赖或单一手段镇痛，转变为以超声引导区域阻滞为基础、联合多类镇痛药的个体化多模式镇痛^[53,77]^。有效疼痛控制既是舒适化医疗需求，也是促进术后心肺脑等系统功能恢复、加速康复的关键，完善的疼痛管理可显著降低术后肺不张、肺部感染发生率及死亡率。核心方案强调MDT协作，围术期全程多模式个体化干预：术前评估疼痛敏感性，予对乙酰氨基酚+NSAIDs预防性镇痛提升痛阈，术中实施区域阻滞从源头阻断疼痛信号，术后以按时非阿片类药物为基础，配合静脉自控镇痛泵，按需少量阿片类药物补救镇痛^[77,90]^。

区域阻滞优先选择覆盖手术区域的超声引导精准阻滞，主要包括胸椎旁阻滞（thoracic paravertebral block, TPVB）、竖脊肌平面阻滞（erector spinae plane block, ESPB）、前锯肌平面阻滞及肋间神经阻滞，均具有良好镇痛效果。胸段硬膜外虽为区域镇痛金标准，但操作复杂、风险较高，已逐渐被精准神经阻滞取代。超声引导区域阻滞效果确切，其中TPVB疗效突出，前锯肌平面阻滞、ESPB、肋间神经阻滞等胸部阻滞疗效非劣于金标准。前锯肌平面阻滞穿刺定位简单，适用于平/侧卧位，针尖受肋骨保护安全性高，可用于复杂疼痛场景的补救镇痛^[91]^。阿片类药物应用趋向“节俭化”，通过充分区域阻滞及非阿片类背景用药减少用量，降低恶心、呕吐、呼吸抑制等副作用^[88]^。

个体化是高质量镇痛的另一核心^[77]^。老年患者肝肾功能可能减退，需调整药物剂量并密切监测呼吸抑制风险；疼痛敏感性高者术前需制定强化多模式镇痛计划，必要时联合疼痛科管理；“无管化”手术（如自主呼吸麻醉）患者需强化区域阻滞，避免影响呼吸。疼痛评估需兼顾静息痛与动态痛，鼓励患者参与评估并根据反馈实时调整方案^[92]^。

疼痛管理全程SDM至关重要。疼痛管理团队术前需向患者说明术后疼痛的必然性与可控性，介绍拟用镇痛策略及可能感受（如阻滞后胸部麻木），帮助建立合理预期，明确疼痛控制目标是支持早期下床、有效咳嗽，而非完全无痛。双方可共同商定可接受疼痛水平[如数字评分法（numeric rating scale, NRS）≤3分]^[88]^，制定以非阿片类药物为主的出院后镇痛计划。通过持续沟通与动态调整，提升患者康复参与度、治疗依从性及满意度^[77]^。

**推荐意见13：推荐GGO术后疼痛管理采用以超声引导区域阻滞（TPVB等）联合对乙酰氨基酚、NSAIDs等镇痛药为核心的多模式镇痛方案，限制阿片类药物使用。镇痛策略需依据患者年龄、肝肾功能、手术方式及疼痛史个体化调整，由MDT疼痛管理团队协作，医患共同确定疼痛控制目标与康复计划，实现安全、舒适、快速康复（2A）。**

GGO术后康复SDM临床路径图见图4。

**图4:**
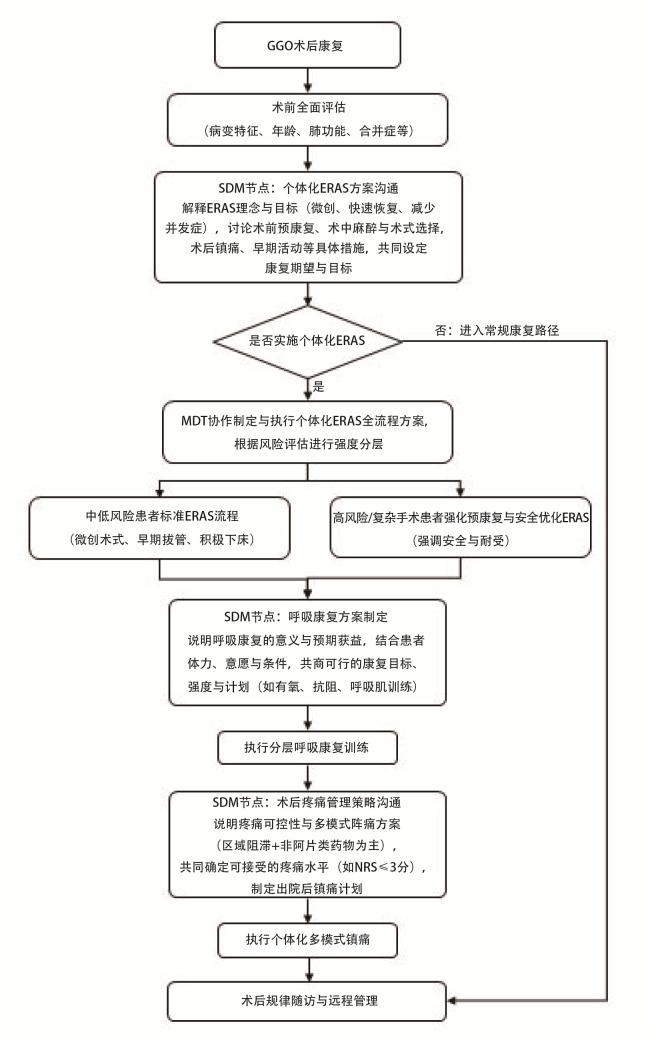
GGO术后康复SDM临床路径图

## 5 术后辅助治疗

GGO术后辅助治疗决策是临床难点，核心争议在于精准识别辅助治疗获益人群，避免对惰性病变过度治疗。与传统实性肺癌不同，磨玻璃为主型肺腺癌预后好、复发风险低，辅助治疗需谨慎^[4]^。AIS、MIA完整切除后几乎无复发风险，共识明确无需辅助治疗^[4,9,93]^。IAC需结合病理分期、高危因素及分子特征进行个体化判断：IA期无高危因素（如微乳头/实体型成分、脉管癌栓、STAS等）者通常不建议辅助治疗；IA期伴高危病理因素（实性/微乳头/复杂腺体成分≥10%）的表皮生长因子受体（epidermal growth factor receptor, *EGFR*）突变患者，Zhang等^[94]^前瞻性APPOINT研究证实，术后予第三代EGFR-酪氨酸激酶抑制剂（tyrosine kinase inhibitors, TKIs）阿美替尼辅助治疗，2年RFS率从75%升至100%，安全性良好，填补该人群治疗空白，为其个体化辅助治疗提供高级别依据；IB期因复发风险存在决策分歧，需综合肿瘤大小、分化程度、胸膜侵犯等评估；II期及以上患者常需辅助治疗，方案依据淋巴结状态及驱动基因突变制定^[93,95]^。

当前深层争议包括：传统肿瘤原发灶-淋巴结-转移（tumor-node-metastasis, TNM）分期对磨玻璃样肺腺癌的适用性局限、分子残留病灶（minimal residual disease, MRD）检测的潜在价值与临床推广难度^[96]^，以及驱动基因阳性（如*EGFR*突变）患者辅助靶向治疗的获益风险比。尤其早期IAC，即便存在敏感突变，仍需慎重权衡低复发风险与靶向治疗的副作用及经济负担^[36,97]^。此外，多发GGO多为多原发病灶，不同病灶驱动基因可能不同，辅助治疗决策需基于最高分期病灶，而非针对所有GGO^[8,41]^。

此决策过程充满不确定性，SDM至关重要^[98]^。医生需全面评估患者复发风险，除病理分期外，还需结合肿瘤CTR、病理亚型、手术切除情况及分子标志物等，向患者清晰说明个体复发风险、辅助治疗的潜在绝对获益、不良反应及经济成本，明确“积极监测”亦是合理选择。尤其临界状态（如IB期、伴高危因素的IA期）患者，需充分沟通了解其复发焦虑程度、治疗副作用耐受性及个人价值取向，共同制定契合意愿与生活质量的方案。MDT讨论为复杂病例决策的重要支撑，整合多学科意见提升决策科学性与全面性^[99,100]^。

**推荐意见14：推荐AIS、MIA患者术后无需辅助治疗（1A）；IA期IAC无高危病理特征或分子异常者，不建议辅助治疗（1B）；IA期IAC行亚肺叶切除，伴实性/微乳头/复杂腺体成分≥10%的** ***EGFR*** **突变患者，经MDT充分评估及SDM，全面告知获益、副作用及经济负担后，可谨慎考虑EGFR-TKIs辅助治疗可行性（2B）；IB期患者需综合评估高危因素及基因检测结果，慎重权衡辅助治疗获益与风险（2B）；II期及以上患者，推荐依据淋巴结状态及驱动基因突变选择辅助化疗、靶向治疗或免疫治疗（1A）。所有决策需基于个体复发风险、患者意愿及生活质量诉求，经医患共同参与及MDT讨论制定，避免过度治疗与治疗不足（2A）。鼓励有条件的中心探索MRD等精准医学技术用于复发风险分层，优化辅助治疗策略。**

GGO术后辅助治疗SDM临床路径图见图5。

**图5:**
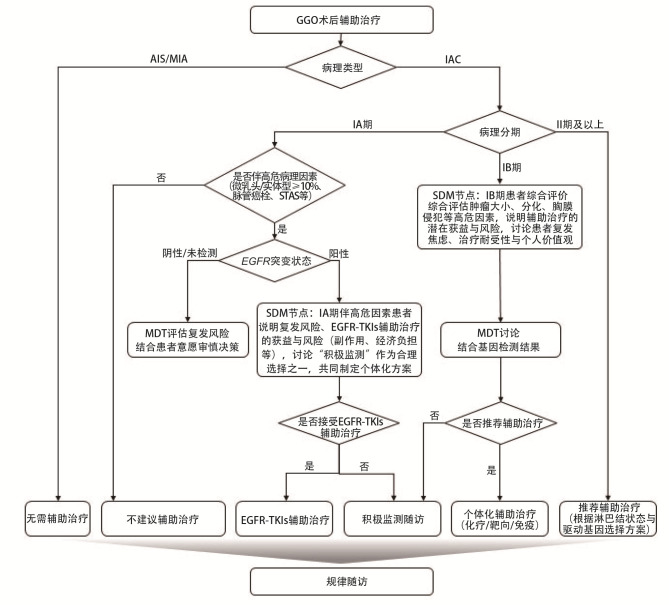
GGO术后辅助治疗SDM临床路径图

## 6 术后随访

### 6.1 随访频率与持续时间的个体化争议

GGO术后随访是长期管理的重要部分，随访频率与持续时间尚无全球统一标准，核心争议是依据个体复发风险制定随访方案，平衡复发监测与过度医疗、心理负担^[9,82]^。随访频率争议集中于固定间隔与风险分层动态调整^[101]^。传统指南多建议术后前2年每6个月复查、后续每年1次，但难以体现GGO患者异质性^[82]^。目前更多观点支持分层管理，高危患者（如IAC伴高危病理亚型、脉管癌栓、STAS或淋巴结阳性）术后早期建议每3-6个月密集随访^[102]^，低危患者（如AIS、MIA或无高危因素的贴壁样为主型I期癌）复发风险极低，可放宽至每年1次甚至更久^[5,36]^。

随访持续时间争议围绕终身随访与有限期随访^[82]^。支持终身随访者认为，肺癌患者始终有第二原发癌风险，部分GGO惰性强，复发可能出现在5年甚至10年后，需长期监测；反对者则认为，AIS、MIA及部分低危I期患者5年后复发概率极低，过度随访成本效益低，还会增加辐射暴露与心理压力，建议5-10年后终止规律影像学随访^[36]^。此外，随访检查手段选择亦有分歧，LDCT辐射低适用于长期筛查，但部分专家推荐高危患者基线及早期随访用标准剂量CT，以获取更清晰图像便于精准对比^[1,82]^。

个体化随访方案需综合病理类型、手术情况、高危因素及年龄等^[102]^。极低危患者（AIS/MIA）可每年随访1次，5年后可商议延长间隔或终止^[36]^；低危I期患者建议至少随访5年，后期酌情放宽间隔；中高危患者需长期甚至终身定期复查^[102]^。多发GGO患者随访需基于最高风险已切除病灶制定计划，同时持续监测未处理次病灶^[8,59]^。年轻低危患者仍建议长期随访，高龄或合并症多者可经充分沟通简化随访流程^[102]^。

术后随访过程中SDM居核心地位。医生需向患者详细说明随访的双重目的（监测复发、发现第二原发癌）及个体化方案的制定依据（病理结果、手术情况、复发风险层级）^[101]^，共同权衡不同策略利弊。密集随访可早发现问题，但增加经济与心理负担；宽松随访减轻压力，但可能略增间隔期复发风险（低危患者该风险极低）^[101]^。同时需尊重患者风险认知与心理承受能力，在医学原则内灵活调整随访计划，并保留根据复查结果动态优化的空间^[101]^。

**推荐意见15：GGO术后随访采用风险分层个体化策略。低危患者（AIS、MIA及无高危因素的贴壁样为主型I期癌）术后1-2年每6-12个月复查，后续每年1次，5年后可酌情延长间隔；中高危患者（伴高危病理特征的I期癌、II期及以上）前2年每3-6个月复查，后续每6-12个月1次，坚持长期随访。所有患者随访以LDCT为主，高危患者基线及早期随访可考虑标准剂量CT。随访计划须经医患充分沟通制定，结合患者临床特点、心理需求及意愿动态调整，平衡科学监测与合理负担（2A）。**

### 6.2 随访检查项目的循证优化

GGO术后随访检查项目优化是临床重要议题，核心是依据个体复发风险制定差异化监测策略，平衡复发发现与过度医疗负担^[9,102]^。共识支持从传统“一刀切”模式转向基于病理类型、分期及高危因素的个体化随访方案。AIS、MIA患者预后极佳，复发风险极低，随访可简化为年度胸部薄层CT，常规行头颅MRI、全身骨扫描或PET/CT等检出率极低，还会增加不必要辐射、经济成本及心理焦虑^[4]^。IAC需进一步区分低危与高危：低危者（如IA期无高危因素）建议术后前2年每6个月复查胸部CT，后续过渡至每年1次；高危者（如IB期及以上，或伴脉管癌栓、STAS、实体/微乳头成分等高危特征）需更密切随访，除定期胸部CT外，可酌情行肿瘤标志物监测、头颅MRI或腹部影像学检查，避免无差别全面筛查^[5,103]^。

确定具体随访项目时需审慎评估各项检查的适用范围与证据支持^[9]^。PET/CT对磨玻璃为主病灶敏感性有限、代谢活性较低，不推荐作为术后常规随访手段^[82]^；肿瘤标志物在早期GGO患者中阳性率及特异性均不高，仅作为高危患者辅助参考^[82]^；支气管镜对周围型无症状病变价值甚微，仅适用于中央型病变或咯血等特定症状时。此外，多发GGO患者随访需基于已切除最高风险病灶的病理结果制定计划，同时长期监测未处理病灶^[8,25]^。随访全程需兼顾“治愈窗口期”对低危患者的意义，及过度检查的生理心理负担，强调随访精准性与成本效益。

SDM居核心地位。医生需向患者详细说明个体复发风险及随访方案制定依据，包括推荐检查与省略的原因，帮助理解“避免过度”与“足够安全”的平衡^[82]^。同时明确随访双重目标（监测复发、发现第二原发癌），教育患者识别新发咳嗽、咯血、骨痛、头痛等预警症状，以便及时就医，无需拘泥于固定复查时间^[82]^。心理焦虑较高或风险认知有差异的患者，需在循证医学原则内尊重其意愿，适当调整随访频率或项目，提升依从性与生活质量^[13]^。随访计划需保持动态灵活，根据每次复查结果及时调整后续策略^[101]^。

**推荐意见16：推荐GGO术后随访检查项目实施风险分层管理：AIS、MIA患者仅需年度胸部LDCT；低危I期IAC患者术后2年内每6个月行胸部CT，后续每年1次；高危患者（IB期及以上或伴高危因素）需更密切随访，除定期胸部CT外可酌情选择肿瘤标志物、头颅MRI或腹部影像学检查，避免常规应用PET/CT、支气管镜或全身骨扫描。所有随访策略须经医患共同沟通制定，结合患者病理特征、心理需求及意愿动态调整，实现科学监测与合理负担的统一（2A）。**

### 6.3 新发病灶的鉴别与处理策略

GGO术后新发病灶的鉴别与处理是随访常见挑战，需结合影像学特征、动态变化及患者个体情况制定策略^[101]^。新发病灶可能为良性炎症、第二原发肺癌或转移复发灶，鉴别要点包括病灶密度、边缘形态、增长速度及实性成分变化等^[5,21,25]^。首次发现、无明显恶性特征的pGGO，建议短期随访（如2-3个月）或经验性抗炎治疗后复查以排除一过性炎症，病灶持续存在但稳定则进入长期密切监测，随访中出现进展（直径增大、实性成分增多）需考虑干预^[3,9,21,25]^。高度疑似转移或复发灶，应启动MDT讨论，依据全面分期、分子检测结果及患者全身状况制定个体化方案（可能包括手术、靶向治疗、放疗或免疫治疗等）^[101,104]^。

决策需充分考虑患者肺功能储备、年龄、合并症及心理状态，尤其既往接受肺切除者，再次手术需谨慎评估肺功能耐受性。同时借助薄层CT、人工智能辅助诊断及影像组学等新技术提升鉴别精准度。SDM至关重要，医生需向患者解释新发病灶的多种可能性，阐明随访意图与安全性，帮助理解“治愈窗口期”下惰性病变的适度观察策略，避免过度恐慌导致不必要干预^[3,13]^。双方共商不同方案利弊，尊重患者对治疗风险与生活质量的权衡，制定符合循证医学原则且体现患者意愿的个体化策略。

**推荐意见17：GGO术后新发病灶首选短期随访鉴别良性病变，持续存在但稳定者建议长期影像学监测，进展性病灶可考虑手术切除（优先亚肺叶切除）或其他局部治疗，疑似转移或复发者需经MDT讨论制定系统治疗方案。所有决策基于病灶特征、患者整体状况及意愿，经医患充分沟通制定，保障医疗安全的前提下避免过度诊疗（2A）。**

GGO术后随访SDM临床路径图见图6。

**图6:**
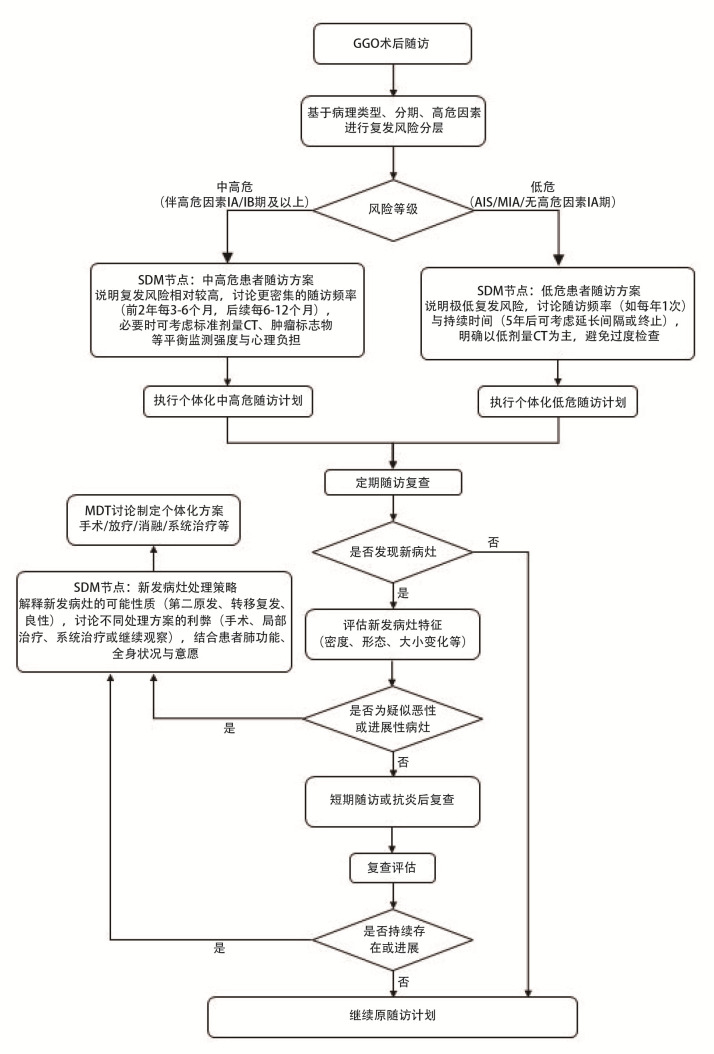
GGO术后随访SDM临床路径图

## 7 总结

GGO诊疗已迈入以“患者价值观为中心”的精准医学新阶段^[2,6,7,10]^。本共识系统回应GGO管理关键争议，首次将SDM确立为贯穿诊疗全周期的核心原则^[12,98]^。SDM超越传统医患关系模式，强调在科学证据与个体偏好间寻求平衡，通过结构化对话将不确定性转化为个体化决策契机^[98]^。

GGO具高度异质性与惰性生物学行为，传统“一刀切”策略过于粗放。本共识明确，从筛查起始年龄、间隔设定，到术中术式选择、术后康复路径，各环节均需融合循证医学与患者生活目标、风险承受力及生命价值观^[6,9,16,80]^。SDM并非让患者独自抉择，而是医患共建决策伙伴关系：医生提供专业研判与证据解读，患者贡献语境化需求与偏好权重，最终形成契合个体的诊疗方案。

为提升SDM可操作性，建议从以下路径与工具推进实施：一是围绕手术时机、术式等关键决策点开展结构化沟通，系统阐释疾病惰性、“转移成功之前阶段”与“治愈窗口期”概念，结合患者影像特征进行个体化风险分层，清晰说明积极监测、亚肺叶切除、肺叶切除及非手术局部治疗等所有方案的预期获益、风险及对生活质量的长期影响；二是开发应用决策辅助工具，如风险可视化图表（附表1，http://www.lungca.org/files/2025s167s1.xlsx）、选择对比卡片、在线进展概率计算器，配发患者健康教育手册^[105]^，并将CTR、生长速率等专业术语转化为患者可理解的比喻与日常语言，避免直接呈现原始数据；三是优化沟通流程，推广“问-答-讲”等技巧，鼓励家属参与，可设立SDM咨询门诊保障沟通时间；四是依托MDT支持系统^[106]^，由护士或个案管理师协助患者教育，探索引入决策教练，医院管理层需将SDM技能培训与实践质量纳入医疗质量评价体系，构建涵盖内容、工具、沟通及系统支持的完整可操作框架。

本共识旨在推动胸外科、呼吸科、肿瘤科等专科在GGO管理中强化协作式、患者价值导向的诊疗模式。预期通过以SDM为核心的结构化沟通与决策流程，提升医疗质量与患者满意度，减少不必要检查治疗及资源浪费，实现医患和谐。

**同济大学附属上海市肺科医院编写委员会**

**执笔专家：**赵晓刚、蔡杰、赵德平

**专家委员会（按姓氏汉语拼音排序）：**包敏伟、鲍熠、陈昶、陈乾坤、陈仰纯、丁嘉安、段亮、何文新、胡学飞、姜格宁、李玉萍、刘鸿程、刘明、刘小刚、鲁铭、吕欣、倪浩、秦雄、任胜祥、史景云、施哲、宋楠、苏春霞、孙希文、万紫微、王海峰、汪浩、武春燕、谢伯雄、谢冬、许亚萍、杨晨路、杨健、杨洋、张雷、张鹏、赵德平、赵晓刚、周晓、朱余明

**特邀审阅（按姓氏汉语拼音排序）：**陈克能（北京大学肿瘤医院）、高树庚（中国医学科学院肿瘤医院）、韩宝惠（上海市胸科医院）、刘德若（中日友好医院）、张国桢（复旦大学附属华东医院）、张昊（徐州医科大学附属医院）、支修益（首都医科大学宣武医院）、钟文昭（广东省人民医院）、周彩存（上海市东方医院）
